# “How Are My Age and Cows Related?” Cognitive Interviewing as a Tool to Pretest Survey Questions in Two Limited Resource Settings

**DOI:** 10.3389/fvets.2022.833748

**Published:** 2022-07-08

**Authors:** Marika Wenemark, Nicholas Ngwili, Dickson Ndoboli, Barbara Wieland, Kristina Roesel

**Affiliations:** ^1^Department of Health, Medicine and Caring Science, Faculty of Medicine, Linköping University, Linköping, Sweden; ^2^Department of Health and Care Development, Region Östergötland, Linköping, Sweden; ^3^Animal and Human Health Program, International Livestock Research Institute, Nairobi, Kenya; ^4^Animal and Human Health Program, International Livestock Research Institute, Addis Ababa, Ethiopia; ^5^Central Diagnostic Laboratory, College of Veterinary Medicine, Animal Resources and Biosecurity, Makerere University, Kampala, Uganda; ^6^Institute of Virology and Immunology, Mittelhäusern Switzerland; ^7^Department of Infectious Diseases and Pathobiology, Vetsuisse Faculty, University of Bern, Bern, Switzerland; ^8^Department of Veterinary Medicine, Institute of Parasitology and Tropical Veterinary Medicine, Freie Universität Berlin, Berlin, Germany

**Keywords:** cross-cultural, cognitive interview methods, livestock, One Health, questionnaire, survey, antimicrobial resistance, behavior

## Abstract

Antimicrobial resistance is a complex topic requiring interdisciplinary solutions embedded in One Health thinking. Currently, many surveys are underway in low- and middle-income countries to study how antimicrobial use in the livestock sector is driving resistance. In a survey, the respondents must understand and answer the questions correctly to produce accurate and valuable results. Pretesting survey questions is therefore important but sometimes not performed due to limited time and resources. Cognitive interviewing is a pretesting method to give insights into the respondent's way of interpreting and mentally processing the survey questions to identify problems and finding ways to improve the questions. It has previously been suggested that cognitive interviews may be difficult to use in some cultural settings. This study aimed to use cognitive interviews in a respondent-adjusted way to study how survey questions related to antimicrobial use are understood and answered by 12 small-scale farmers in Kenya and Uganda. The results show that even a small number of interviews and using interviewers with limited knowledge of cognitive interviewing can identify many problems in survey questions and the survey tool. Cognitive interviews may provide a feasible and affordable way of pretesting questionnaires in situations where time and resources are limited, for example, during a disease outbreak.

## Introduction

One Health is an integrated, unifying approach that aims to sustainably balance and optimize the health of people, animals, and ecosystems (1). In contrast to Veterinary Public Health at the interface between animals and humans with human health as the key outcome, One Health acknowledges that human, animal, and environmental health are closely interlinked and connected and need to be looked at as a system. As such the environment does not only impact disease spread (i.e., climate change shifting the distribution of vector-borne diseases) but is also equally affected by the increased population growth and consequently, the increased demand for animal protein (i.e., greenhouse gas emissions, pollution). The increase in humans and animals adds pressure on resources and means more treatment of infectious diseases with antimicrobials including antibacterials, antifungals, antiparasitics, and antivirals. These substances are often used across species and can end up in the environment.

Antimicrobial resistance (AMR) is one of the major global health threats, projecting that as many as 10 million people could die annually from AMR by 2050 (2). A more recent systematic review estimated 4.95 million deaths associated with bacterial AMR only in 2019, including 1.27 million deaths directly attributable to bacterial AMR (3). These estimates indicate that bacterial AMR is a health problem with a magnitude similar to major diseases such as HIV and malaria, with the highest regional burden in the Sub-Saharan African region (3). The livestock sector is often held responsible for disproportionate use of antimicrobials to either promote growth or mask hygiene and biosecurity issues on farms. However, one of the major gaps in controlling AMR in Sub-Saharan Africa is the lack of data and knowledge on behavioral drivers for the use of antimicrobials and how they can be addressed. This data gap prompted the development of the AMUSE tool, a survey tool to assess antimicrobial use in livestock systems in low- and middle-income countries.

The common way of pretesting questionnaires in biomedical surveys at the International Livestock Research Institute (ILRI), for instance, risk factor analyses which relate a lot to practices that lead to exposure to zoonotic diseases, is as follows: First, the researcher goes through the questionnaire with peers to speak out the questions. Many peers in the field of research for development in Africa are livestock keepers or crop farmers and therefore, represent a knowledgeable test group. Speaking the question out loud often leads to rephrasing questions to avoid nested sentences, leading questions, or several questions asked in one. Following that, the questionnaire is administered to some sample farmers who represent the target group. Mostly, the target audience is rural small-holder farmers with primary education and multiple agricultural activities to provide for their families' livelihoods and food security. In many African countries, there is one official administrative language, such as English, French, Kiswahili, or Portuguese, and the questionnaire is written and administered in that predominating administrative language assuming that the respondent has enough knowledge to understand and respond to the questions. If that is not the case, the interviewers, who are usually nationals of the country where the survey is implemented, translate the questionnaire on the spot (or on the fly), or the respondent understands the questionnaire in the official language but responds in their local dialect. During this pretesting step, the researcher usually monitors how much time it takes to administer the questionnaire and notes if the respondents ask questions about how to answer specific questions. This process however gives little insights into how questions are understood and answered by the respondents. We cannot be sure if, for example, the respondent has the same understanding of an issue as was intended when developing the question.

In a survey, the respondents must understand and answer the questions correctly to produce accurate and valuable results. Cognitive psychology provides a theoretical framework to understand how respondents answer survey questions (4, 5). The four steps needed to answer a question are (a) comprehension of the questions, (b) retrieval of the information asked for, (c) judgment of the information, and (d) response. Problems in comprehension are, for example, if the question is understood in a different way than intended or if different respondents understand the question in different ways. Problems in retrieval occur when the respondent cannot remember or does not have access to the information asked about. Problems related to judgment have to do with the processing of the information to formulate an answer, for example, deciding if a recalled event should be included in the answer or combining different experiences to an overall attitude. Finally, the respondents must respond to the question by choosing between the response options provided or by formulating their response to an open-ended question. Failure to perform any of those steps can result in incorrect or unprecise answers.

Cognitive interviewing is a questionnaire pretesting method to give insights into the respondent's way of interpreting and mentally processing the survey questions (6–8) in national surveys as well as cross-cultural settings (9). Understanding what causes problems for respondents often gives insights into how to improve the questions (7). Cognitive interviewing is a qualitative method that has developed over the last 30 years. One of the most commonly used techniques to perform cognitive interviews is think-aloud interviewing in which the respondent is asked to speak out everything (s)he is thinking when answering the questions. This method is often combined with asking specific questions (probes) to give deeper insights into the thought process. The strength of cognitive interviewing is the insight into the response process with the aim of both improving the quality of data but also improving the survey tool from the perspective of the respondent (10). Limitations are, for example, that it is a qualitative method that will not give numbers or show the extent of the problems identified, it relies on the participants' ability to verbally articulate their thoughts and the results are often based on a small number of interviews (7).

It has previously been suggested that cognitive interviews work differently in different cross-cultural settings (11–15). For example, it may be difficult for respondents in Asian settings to express critical views of the questions, especially if the survey is perceived as a representation of an authority (14, 16). Pan (14) identifies several challenges of performing cognitive interviews in Chinese: The first is explaining that the purpose of the interview is not to test the participant but to test the questionnaire; the second is that the participant is trying to find “the right” answer to satisfy the interviewer; and the third is related to problems with the respondent understanding and answering probes (especially paraphrasing types). Other authors have reported similar results when performing cognitive interviews in the Korean language, in Japan, and among Chinese immigrants in the United States (17–19).

In Africa, there are examples from Ethiopia, Kenya, Malawi, Zimbabwe, and Zambia of conducting cognitive interviews with different success rates (20–22). Several authors have reported respondents being anxious and uneasy during interviews in populations not used to taking part in surveys (15, 20, 23). In some cases, the researchers asked several probes after each survey question, and this made the respondents feel uncomfortable (20). However, after the interviewers abandoned the highly structured probing and allowed for more flexibility in the interview, the situation improved.

Another challenge of carrying out cross-cultural surveys or surveys in countries with many local languages is that in many situations, it is not possible to do full-scale multilingual translations. In practice, according to our experience, many studies use on-the-fly translations by local interviewers. Such translations require the questions to be clear and specific to make the task easier for the interviewer and reduce the risk of questions not being accurately and consistently asked, leading to bias in the answers (24). Using a questionnaire in cross-cultural settings also restricts the possibilities for extensive training of the interviewers in cognitive interviewing to be able to pretest the questions in many different languages.

It is a good scientific practice to pretest questionnaires and survey tools (25–27). However, it may not be possible to use state-of-the-art techniques due to the number of different cultural settings or languages and the lack of institutional capacity or time. In cases of natural disasters or disease outbreaks, there may be, for example, very limited opportunities and time for pretesting. However, also in studies where pretesting should be possible, it is often not prioritized. In a recent audit of survey pretesting in a sample of medical education journals, <7% described pretesting of survey items before use (28). In the same study, the authors conclude that the low frequency of pretesting was the same when comparing articles published in low- and high-impact factor journals. Considering the meticulous methods described in the literature, researchers may be discouraged from doing even simple pretesting.

In the context of AMR, which is a very complex technical subject area on its own, there is a need for pretesting methods that can be used with limited resources, including time, but still, allow one to detect problems in the questionnaire design. While the approach of cognitive interviews for pretesting is not novel, it is not commonly used in low-resource settings. In this study, we show how cognitive interviews can be used feasibly and affordably in situations where there is a need to, for example, do so quickly during a disease outbreak or when the pretests must be performed in several local languages.

## Materials and Methods

### The Survey Tool Used for Pretesting

The AMUSE tool was developed in 2018 by a team from the livestock health flagship of the CGIAR Research Program on Livestock to investigate the key linkages in the AMR conceptual framework (29). Following a review of different survey tools used in the past, the team drafted the first version of the AMUSE questionnaire, which was launched in several countries in Africa and Asia (30) including Ethiopia (31), Uganda (32, 33), and Vietnam (34). As a next step, the questionnaire was developed into a generic tool to assess antimicrobial use in animal production (including livestock, poultry, and fish) to enable comparison between different countries and settings (Supplementary Material). The original version of the questionnaire consisted of 76 questions, sometimes with several items per question. There was no ambition to suggest improvements for the questions beforehand in this study since the task was using the cognitive interview approach to pretest the existing version. The cognitive interviews were done on the entire questionnaire to pretest all questions as well as question order and length.

### Setting and Sample

Interviews were conducted in March 2019 in Murang'a county (Kandara subcounty) and Kiambu county (Kikuyu subcounty) in Kenya (*n* = 7), and in Mukono district (Mukono subcounty) and Nakaseke district (Kapeka subcounty) in Uganda (*n* = 5) by two interviewers, one a Kenyan and the other a Ugandan. Both interviewers were men, in their early thirties, and had worked in the field of livestock research for approximately 10 years including interviewing experience. Four notetakers (two in each country) were responsible for taking notes during the interviews. An experienced survey researcher was present during the fieldwork who did not interfere in the interview but made observations on the process and perceived non-verbal signs. After each interview, the group had a debriefing about the experiences from the interview and the interviewers were advised for the upcoming interviews.

The participants were selected purposively with the help of local contact persons. We asked for livestock farmers of different socio-economic backgrounds, livestock species maintained, and a balance between male and female farmers. We interviewed seven men and five women aged between 28 and 68 years and with a variation in urban and rural settings. The participants were small-scale farmers with typically 3–15 animals (cows, pigs, goats, or chickens). The interviews were conducted in Kiswahili in Kenya and Luganda in Uganda, except for one in English.

The interviewers and notetakers were given a half-day introduction to the cognitive process of answering survey questions and the use of cognitive interviews to get insights into the response process of a respondent. They were trained to introduce the task of “think-aloud” to respondents, what to note during the interview, and how to probe. A few predefined probes were used in all interviews and the interviewers were encouraged to use spontaneous probes according to their judgment. The two notetakers were trained to make notes on a standardized protocol, especially noting things that would not be caught on the recordings such as showing signs of being uncomfortable or getting tired. They were supposed to be silent observers but were allowed to add probes at the end of the interview if they made an observation that suggested that a question was misunderstood or if there was something else that the interviewer did not follow up on.

It was important for the interviewer to contribute to a respondent-adjusted approach by creating a friendly, relaxed atmosphere to make the participant feel comfortable because we wanted to understand how the respondent processes the question instead of solely focusing on the response to the survey question. This was done by some small talk when looking for a good spot to do the interview and avoiding any types of authoritarian or bureaucratic approaches. The time limit of the interviews was set at 1 h and they lasted between 45 and 60 min. To thank the participants, in-kind incentives of 500 g of sugar and a packet of tea were given in Uganda, and 500 g of sugar and 2 kg of maize flour were given in Kenya, but not announced in advance. None of the interviewed farmers lived close to each other or knew each other to ensure that information about the token of appreciation was not spread to subsequent respondents and thereby avoiding the risk of the incentive influencing their choice to participate. The interviews were done during 1 week in each country, and all interviews were audio-recorded after having obtained the participants' consent.

### Data Management and Coding

One of the two notetakers did a simultaneous translation into English and transcription of the audio-recorded interviews as soon as possible after the interview (usually the same or the next day). The transcriptions were then re-organized to gather all findings of a specific question. All findings were then categorized into (1) cognitive problems (problems in comprehension, retrieval, judgment, or response) or (2) other problems (unclear relevance, inappropriate assumptions, sensitive questions, problems in translation, phrasing, or other interviewer mistakes) (Table 1). The findings of each question were then analyzed and suggestions were made on how to improve the questionnaire. In the Results section, citations from interviewers (I) and participants (P) are given. They are sometimes shortened but not altered in other ways.

**Table 1:** Categories of problems with the questions and description.

**Category of problems**	**Description**
**Cognitive problems**	
Comprehension	Problems in understanding the question or specific concepts
Retrieval	Problems in retrieving or recalling the information asked for
Judgement	Problems in estimating, calculating or making a judgement
Response	Problems in formulating or selecting an appropriate answer
**Other problems**	
Unclear relevance	Respondent does not understand the reason for the question
Inappropriate assumptions	Question assumes things that are not true for the respondent
Sensitive question	Respondent perceives the question as sensitive or intruding
Interviewer rephrasing question	Interviewers use different or incorrect phrasing of question
Interviewer mistake	Interviewers' mistakes, for example, in filter questions or reporting of answers

## Results

The complete original AMUSE tool with 76 questions was administered to 12 respondents using a cognitive interview approach. The findings were consolidated for all questions in a comprehensive working report that was handed over to the team working on the development of the AMUSE questionnaire who then developed a revised version of the original questionnaire. The cognitive interview team did not take part in the revision, which resulted in a new version of the questionnaire (30). The working report can be shared upon request.

The following sections summarize how the method worked in practice and gives specific examples of questions from the original questionnaire and how cognitive interviewing helped identify problems in the design of them as well as suggested revisions based on the analysis.

### Experiences of How the Cognitive Interview Practice Worked From the Respondents' Perspective

In general, the use of a respondent-adjusted interview style worked well. Respondents were relaxed and most of them engaged in the think-aloud process. Some respondents were quieter and were asked more probes to compensate for this. In some cases, the interviewer would just say “mm” or “aha” and keep looking at the respondent as if expecting more comments and giving enough time for the respondent to continue before moving to the next question. This was a successful way to give the respondents time to reflect and encourage them to talk more without the pressure of probes. Signs of the respondents feeling comfortable and interested were that they, for example, questioned the relevance of questions and commented on questions that seemed redundant or the length of the questionnaire. No negative reactions of respondents such as feeling uneasy, uncomfortable, or distressed were observed.

### Examples of Findings and Suggested Revisions for Specific Questions

Example 1: Do you have hired workers on your farm? Yes / No, family members only

I: Do you hire workers on your farm to look after your animals? P: I had them in the past, but these days I don't. I do the work myself. Maybe occasionally I can pay a little money to a casual laborer to help me do some work.

The cognitive interviews revealed two important things. In several cases, the interviewer spontaneously added information that the question concerns work with the animals. This need to be specified in the question if the answer should not include workers that only help with, for example, crops. The interviews also showed problems with comprehension of the concept “hired workers.” As shown in example 1 the participant did not include casual laborers. The question needs to be rephrased to clarify the intention to include all kinds of hired workers, even just temporary paid help. A new suggestion could be: Do you have employees or casual workers that are involved in working with the animals?

Example 2: Was the disease diagnosed other than by yourself? Yes / No. If yes, by whom? Traditional healer / Community animal health worker / Private veterinarian (Diploma, BVM), Official (governmental) veterinarian / Other (This question is a follow-up question after a question about what diseases the different species had in the last 2 weeks).

P1: Which disease? I: The diseases that the pigs and poultry are facing now. P1: In pigs. I called a vet.

P2: A private vet. I: So this vet, do you know his level of education or his qualifications? P2: I measure qualification from curing my animal [laughing] as long as he treats it and it gets cured and even a second time he cures them why don't I call him a vet.

The structure of the questionnaire was to ask each question for all the different animal species on the farm. The interviews showed that it was burdensome for both interviewers and respondents to go through each question for each animal species. For example, for the interviewer to ensure if the answers covered all species or not. In example 2, P1 had experienced diseases in the pigs and poultry and when the follow-up question was asked on who diagnosed the disease (s)he is not sure which disease the question concerns. This complicated structure often required the interviewer to ask extra questions to ensure all species had been covered and, in some cases, caused missing or incorrect registrations of answers. To avoid those problems, a suggestion from the results of the interviews was to organize the questions into one section for each species. In that way, it would be easier for the farmer to focus only on one species at a time and answer all questions about the diseases experienced and the drug used, for example, in the pigs.

Some questions were asked about things that the farmers had limited knowledge of, and therefore could not give an accurate response. This is usually referred to as retrieval problem when the respondent has no knowledge or cannot remember the information asked for. One such example is when asking about qualifications of the veterinarian in example 2. On another question that asked about the qualifications of veterinarians, one farmer explained the difficulties like this: *P: No one will present their certificate or anything like that. We hear about the doctors from word of mouth, like the one I have engaged with the longest was introduced to me by a friend*. Similar problems occurred in a question about what drugs had been used on their animals by veterinarians. Two of the participants described this as impossible for them to know since veterinarians sometimes concealed the drugs. *I: Can you remember any name of a drug you or your vet has used? P1: No, the vet never allows me to have a look. P2: I never got to see them from the vet, they are always in a bag*.

Example 3: Which period of the year do you regularly sell pigs? Throughout the year / Seasonal (possibilities to mark specific months from January to December)

P1: I sell after six months. Sometimes I sell the piglets at 2 months old when I need money urgently. But I would prefer to sell when 1 year old. This way I can profit more. In case of urgent money, I sell at 2 months.

P2: I sell whenever there is demand, if a buyer comes, I will sell the pigs no matter the season. I cannot refuse to take money.

The questions about what periods over the year the farmer sells milk, eggs, or animals caused problems for several farmers since considerations for selling include, for example, opportunities to sell, shortage of feed, or the need for money. The question incorrectly assumes that selling is predominantly done during certain periods of the year which causes problems for the farmer to select an appropriate response option. More suitable response options could be: Throughout the year / Certain months or periods / Occasionally.

Example 4: Livestock contributes to: To half or more of the household's income / To less than half of the household's income / Does not contribute to the household income.

I1: What amount of the income does it contribute to?

I2: To what extent does farming contribute to your total income?

I3: How much do poultry and cattle contribute to your income?

I4: The general income – what's the percentage that is contributed from the livestock?

This example shows that when the information asked for is not stated as a question, the interviewer needs to transform the text into a question. This was the case in several questions in the questionnaire and caused an additional burden for the interviewer to rephrase the text into a question and simultaneously make the translation. It caused unnecessary variations of phrasings of the question in each interview. In the example, the question was phrased as *What amount? To what extent? How much?* and *What percentage?* There is also a variation if referring to income from farming or specifically from the animals and none includes information that the question concerns the income for the whole household. A better question could be: What part of your household's income comes from the animals?

Example 5: Age of the respondent (years).

I: How old are you? P: 41. Maybe it will depend on one's understanding, because when you approached me you talked about cows, so one can wonder how my age and cows are related. I: So, one can wonder how the question is related to animals. P: Yes.

Again, the text needed to be rephrased into a question by the interviewer. One of the participants also asks about the relevance of a question in relation to the purpose of the survey. This version of the questionnaire started with eight questions about the characteristics of the respondent, the household, and the farm. For example, the question in Example 4 about the household income was asked before any questions about what animals the farm had. It was therefore suggested from the interviews to start asking about the animals as this was specified as the purpose of the interview and would probably be expected, and relevant questions from the respondents' perspective and the demographic questions were moved to the end of the questionnaire.

Example 6: What do antibiotics do? (multiple answers possible) Cure sick animals / Prevent animals from becoming sick / Cure sick animals and prevent animals from becoming sick / Fattening

I1: So, what of antibiotics what do you think they do? P: they kill sickness.

I2: And what are antibiotics for according to you? P: according to how I understand them, they are for curing.

The question “What do antibiotics do?” led both interviewers to rephrase it a bit softer than the question stated in the questionnaire. The interviews also showed a missing response option. It was not possible to register “Don't know” even if a respondent specifically expressed, *I really don't know*.

For most check-all-that-apply questions in the questionnaire (like the one in Example 6 allowing multiple answers), it was not specified if the interviewer should read all options or just tick the ones the respondents mentioned spontaneously. This sometimes led to missed information, for example, when all species of livestock were not read out, chicken kept on a farm with mainly pigs were not mentioned. In other cases, the information would differ substantially depending on if the interviewer read all alternatives that the participant could choose from, or not. In Example 6, in some cases, the interviewer asked specifically about the options not mentioned which resulted in more registrations compared to answers based only on what the participant spontaneously mentioned.

Because it is a tick-all-that apply question, there should be no need for the response option “cure sick animals and prevent animals from becoming sick.” Suggestion for the revision was to re-formulate the question to make it clear that the respondents should answer what they believe. A further suggestion was to make it clear that each option should be read out. For example, saying What do you think antibiotics can be used for? Followed by three sub-questions; Do you think antibiotics can be used to cure sick animals? Yes / No / Don't know and likewise for “to prevent animals from getting sick” and “to make animals grow faster.” This will give information about the farmers' understanding of each of the uses and which uses they think they know about, and which they do not know.

Example 7: Herd flock size (number of animals for each species). Pigs: Sows / Boars / Growers or fatteners / Piglets (<3 months).

P: In total I have 10 pigs, with piglets inclusive. I: How many sows do you have? P: Six. 1 adult and 5 piglets. I: How many young pigs do have? P: 5 female ones. I: How many under the age of 3 months? P: Three, no, five I: Five? P: Yes.

The cognitive process becomes complicated when starting to divide all animals into gender and then divide into adults and young ones and then finally add the young male and female together. There are 10 pigs in total, but only 1 sow and 5 piglets are registered (probably because of the confusion about the number of female pigs where the farmer counts also the female piglets). Based on the results from the interviews, the suggestion is to make the question easier by first asking only about adults above a certain age. Out of those adult animals you just mentioned, how many are sows. Then continue to ask about the younger animals. This would probably make it easier for the participant to get it right from the start and less need for extensive probing for the interviewer.

Example 8: Do you consume milk from animals that were just treated with antimicrobials? (Similar questions for eggs and meat).

I: So, the cow that has been on antibiotics can you take its milk? P: For the first 3 days we give it out. I have a friend who has pigs, so I give the milk to the pigs. I: So, what of the poultry can you eat its eggs when it has been on antibiotics? P: Yes, we do sell them. I: Okay but even you at home do you eat it? P: But for us we don't normally eat eggs I: Let's take an example that you eat eggs, can you eat them? P: Yes, we can because even people take antibiotics but for the cow the medicine that treats fever in cows - people don't use it. So that's the difference.

Although this is a sensitive question some farmers were open about consuming and selling products. But on the other hand, some farmers said they would always dispose of the milk or eggs but when asked about how honest other farmers would be they thought many would not be honest.

I1: Do you think most farmers will give honest answers to this question? P: Most people will lie.

I2: Will they [other farmers] be honest when asked about this? P: The dairy farmers will lie, because pouring out milk is hard for many. You know why I say this is since someone might have a lot of milk on their farm and they will not be willing to pour out a significant amount of this.

These examples show that the cognitive interviews identified various problems from almost all categories listed in Table 1.

### Example of How the Cognitive Interview Practice Worked With Interviewers With No Former Experience in Cognitive Interviews

Interviewers who are not familiar with performing cognitive interviews may encounter problems especially when it comes to when and how to probe. In some cases, the analysis showed unclear statements by the farmer that an experienced cognitive interviewer may have identified and followed up with a probe. There were also situations when the interviewer did not probe neutrally.

I: So, do you have some hired employees at the farm? P: Yes, we have and even have people from outside. I: And those people from outside are they friends? R: They are casual laborers I: But they are also hired? P: Yes, they are.

A more optimal probe after the participant's first answer could be “Can you tell me more about that?” or “Can you tell me more about what you mean by people from outside?” instead of suggesting them to be friends. This shows the importance of transcribing the interviews to be able to take the phrasing of probes into account in the analysis. In the above example, the probe is not optimal but still results in valid information. The respondents describe his/her definition of “people outside” and were not affected by the interviewer's suggestion that they were friends.

## Discussion

We wanted to explore the usefulness of cognitive interviewing in a situation that would probably be the case in multinational surveys involving many different settings and local languages and with limited funds for translations “by the book” and for pretesting. Pretesting by carrying out a few cognitive interviews can help identify problems that can later be avoided in real survey interviews. The results from the interviews led to a major revision of the questionnaire (30).

The interviewers in this study were experienced in conducting interviews but had no previous experience with cognitive interviewing. The interviewers need to understand that their role in cognitive interviews is different from that in a study where the task is only to register answers. In a cognitive interview, the interviewer needs to be sociable, communicative, and able to identify what issues to follow up by probes and to know when to proceed depending on the respondent's mood and reactions. Mohorko and Hiebec (35) have discussed the importance of interviewer involvement for the successful results of cognitive interviews.

Other studies have found that certain types of probes may be difficult for respondents, especially paraphrasing probes or too many probes (14, 20). A possible explanation given by Pan (14) is that Chinese students are taught to memorize and repeat texts and not express opinions or challenge authorities. Martin et al. (20), with experience in Kenya and Ethiopia, suggest difficulties due to participants being unaccustomed to thinking aloud and answering cognitive probes. On the contrary, Vreeman et al. (23) reported that respondents in Kenya have an easier time answering probes than “thinking aloud” and suggest it may be due to a cultural communication style that values listening higher than verbalizing thoughts. Participants in that study said that they felt disempowered and blamed themselves for not being able to answer due to lack of education. In the second round of cognitive interviews, the instructions were clarified, the number of probes reduced, and difficult probes such as paraphrasing were avoided. Another study shows that the think-aloud method does not work in some cultural settings such as India, but the cognitive interviews still revealed extensive question failures (15). In this study, we balanced the type and number of probes to make the respondent feel comfortable and competent; we found that this flexible approach to the interviews resulted in valuable information. For example, just saying “mm” or “eeh” and waiting for the respondent to keep talking made it less necessary to ask specific probes and thereby avoiding questions that could be perceived more like an interrogation for an insecure respondent.

The relaxed atmosphere that the interviewers managed to establish was probably a crucial factor in the successful use of cognitive interviews in this study. In earlier studies on cognitive interviews, distress among respondents was observed and the authors noted the importance of cultural adaptation (19, 20). We find, however, little information on how to train interviewers to build trust and create a friendly situation in cognitive interviews as is done, for example, in participatory epidemiology (36). In this study, for example, interviewers made small talk with the respondents while looking for a good spot in the shade to conduct the interviews. The interviewers were also asked to wear clothes that are not too formal, and avoid such as a lab coat or suits and ties that could imply a visit by an authority figure. Based on the authors' experiences from working on different projects in the area, many rural smallholder farmers have little exposure to formal surveys, also they are humble, often have little trust in governments, and may feel audited if a formally dressed investigator asks questions. For instance, pastoralists may be reluctant to respond to questions about their accurate herd size out of fear of taxation. Another probably crucial factor was the introduction given to farmers that the questionnaire had been made for use in another country and the purpose of this interview was to find out if the questions were possible to use in Kenya and Uganda or if the questions needed to be adjusted for the new setting and population. The participants were also asked for advice on what they would change about the questionnaire or specific questions. This probably made the participants feel good in a way they could contribute to making the questionnaire better as well as less prone to perceive the situation as a test or investigation. Suggestions to improve the questionnaire included making it shorter, avoiding redundant questions and “elaborating on the questions so that one can easily understand.” These suggestions show that the participants felt comfortable raising points of criticism.

The cognitive interviews in this study gave many and varied results that helped revise the questions. This is particularly important, as the questions addressed different concepts, such as knowledge, behavior, and questions on context and disease problems. We believe that the method used in this study identified the most significant and common problems and provided valuable information to revise the questions before further use. Cognitive interviews are often used in combination with other pretesting methods such as focus groups, usability testing, and pilot tests. The strength of cognitive interviews is that they have the potential to reveal problems such as misunderstanding of questions that would, for example, in a pilot test seem to be a valid answer. On the other hand, a pilot test based on a larger number of respondents can reveal, for example, problems with ceiling effects and item nonresponse (7).

One limitation in this study was that the interviewers did not always succeed in identifying problems that would have been clarified by one or two probes. They would also sometimes ask probes in a leading instead of a neutral way as shown in Section 3.3 of the Results. However, also when the probes were not optimal, the results still provided valuable knowledge.

The questionnaire was also too long to provide a deep understanding of the cognitive process by using probes for each question. It was, however, still possible to identify several problems that needed clarification in the revised version of the questionnaire. Furthermore, the interview recordings were translated and transcribed simultaneously. An alternative procedure could have been to transcribe it first in the local language and then translate it into English. That would, however, take more time and could lead to more errors when the information is going through two separate steps of transcription and translation. To conduct cognitive interviews with limited resources, we think the simplified process with simultaneous translation and transcription worked satisfactorily.

Another limitation was that we only conducted a single round of 12 cognitive interviews. What is an appropriate number of interviews depends on issues such as complexity of the questions and the diversity of the target population (7). We had aimed for 5–10 interviews in each country since it is often advised to do 5–15 interviews per interviewing round (8). All interviews were done with smallholders in low- and middle-income settings. The purposive sample led to both male and female respondents and different livestock species being kept to ensure a variety of perspectives. The findings were summarized in a working report which was handed over to the team working on the development of the AMUSE questionnaire. It would have been valuable to make another round of interviews after the revision to pretest also the revised version of the questions as well as continuous testing in other languages and cultural settings.

Because we wanted to evaluate how the interview method worked, we used two notetakers that would also observe the atmosphere during the interview and note any signs of the respondents feeling uncomfortable or irritated. In an ordinary pretest we believe one notetaker would be enough. It was however very valuable that the notetaker who participated in the interview also made the transcription. In this study, a survey researcher with long experience in performing cognitive interviews was present in the field to follow the process. We believe that it is important to give interviewers with limited experience in cognitive interviewing possibilities to discuss any problems they encounter and get feedback on the results of the first interview before proceeding. This can, however, probably be done virtually. Despite the differences in cultural background and previous experience in cognitive interviews, all participants in the research team agreed that the method was successful in finding various problems with the questions and perceived that the respondents felt comfortable and at ease during the interviews. Scott et al. (15) discussed difficulties in interviewing respondents one-on-one without family members present. This was a minor problem in this study as the questions were not sensitive to answer in the presence of family members. However, there were local government veterinarians who were eager to participate in some of the interviews. This could distort the results when questions relate to information that farmers would not want to reveal to their local veterinarian. It is important to give interviewers clear instructions on how to handle such situations and provide them with responses to explain why external persons cannot participate.

## Conclusions

The results show that using cognitive interviewing, even with a small number of interviews and using interviewers with limited knowledge of cognitive interviewing, can identify many problems in survey questions and the survey tool. Cognitive interviews may provide a feasible and affordable way of pretesting questionnaires in situations where time and resources are limited, for example, during a disease outbreak.

## Data Availability Statement

The raw data supporting the conclusions of this article will be made available by the authors, without undue reservation.

## Author Contributions

MW conceived the study, design and implementation of interviews, and wrote the manuscript. KR conceived the study, design and implementation of questionnaires, and revised the manuscript. NN and DN conducted the fieldwork. BW co-developed the AMUSE tool and revised it following this study. All authors contributed to the article and approved the submitted version.

## Funding

The study was funded by the CGIAR Research Program on Livestock led by the International Livestock Research Institute (ILRI) and the CGIAR Research Program on Agriculture for Nutrition and Health led by the International Food Policy Research Institute. The research was implemented under the CGIAR Antimicrobial Resistance Hub hosted by ILRI. The open access publication fee was provided by the CGIAR Initiative-Protecting human health through a One Health approach. We also acknowledge the CGIAR Fund Donors (https://www.cgiar.org/funders). NN was funded by the German Academic Exchange Service (DAAD) through an in-region PhD fellowship in partnership with ILRI.

## Conflict of Interest

The authors declare that the research was conducted in the absence of any commercial or financial relationships that could be construed as a potential conflict of interest.

## Publisher's Note

All claims expressed in this article are solely those of the authors and do not necessarily represent those of their affiliated organizations, or those of the publisher, the editors and the reviewers. Any product that may be evaluated in this article, or claim that may be made by its manufacturer, is not guaranteed or endorsed by the publisher.

## References

[1] Joint FAO/OIE/UNEP/WHO One Health High Level Expert Panel (OHHLEP). One Health definition developed by the OHHLEP states. (2021). Available online at: https://www.oie.int/en/tripartite-and-unepsupport-ohhleps-definition-of-one-health/ (accessed June 23, 2022).

[2] O'Neill J. Tackling drug-resistant infections globally: final report and recommendations. London: Review on Antimicrobial, Resistance (2016). Available online at: https://amr-review.org/sites/default/files/160518_Final%20paper_with%20cover.pdf (accessed June 23, 2022).

[3] Antimicrobial Resistance Collaborators. Global burden of bacterial antimicrobial resistance in 2019: a systematic analysis. Lancet. (2019) 399:629–55. 10.1016/S0140-6736(21)02724-0 35065702 PMC8841637

[4] Tourangeau R Rips LJ Rasinski K. The Psychology of Survey Response. Cambridge: Cambridge University Press (2000).

[5] Tourangeau R. Cognitive aspects of survey measurement and mismeasurement. Int J Public Opin Res. (2004) 15:3–7. 10.1093./ijpor/15.1.328205551

[6] Beatty PC Willis GB. Research synthesis: the practice of cognitive interviewing. Public Opin Q. (2007) 71:287–311. 10.1093/poq/nfm006

[7] Collins D ed. Cognitive Interviewing Practice. London: SAGE Publications Ltd (2015).

[8] Willis GB. Cognitive Interviewing: A Tool for Improving Questionnaire Design. Thousand Oaks, CA: Sage Publications (2005).

[9] Willis GB. Research synthesis the practice of cross-cultural cognitive interviewing. Public Opin Q. (2015) 79:359–95. 10.1093/poq/nfu09229026639

[10] Wilson L Dickinson E. Respondent Centred Surveys. Stop, Listen, and Then Design. Thousand Oaks, CA: Sage Publications (2022).

[11] Guss CD. What is going through your mind? Thinking aloud as a method in cross-cultural psychology. Front Psychol. (2018) 9:1292. 10.3389./fpsyg.2018.01292 30150948 PMC6099082

[12] Hannan A. Heckert J. James-Hawkins L. Yount K. M. Cognitive interviewing to improve women's empowerment questions in surveys: application to the health and nutrition and intrahousehold relationships modules for the project-level Women's Empowerment in Agriculture Index. Maternal and Child Nutrition. (2020) 16:12781. 10.1111./mcn.12871 31288300 PMC7038906

[13] Huang KT Owino C Vreeman RC Hagembe M Njuguna F Strother RM. Assessment of the face validity of two pain scales in Kenya: a validation study using cognitive interviewing. BMC Palliat Care. (2012) 11:5. 10.1186/1472-684X-11-5 22512923 PMC3393614

[14] Pan Y. Cognitive interviews in languages other than English: methodological and research issues. American Association for Public Opinion Research conference, Phoenix, AZ. (2004) 13–16.

[15] Scott K Gharai D Sharma M Choudhury N Mishra B. Yes, no, maybe so: the importance of cognitive interviewing to enhance structured surveys on respectful maternity care in northern India. Health Policy and Planning. (2020) 35:67–77. 10.1093/heapol/czz141 31670773 PMC7053388

[16] Chan AY Pan YL. The use of cognitive interviewing to explore the effectiveness of advance supplemental materials among five language groups. Field methods. (2011) 23:342–61. 10.1177/1525822x11414836

[17] Jang MK Kim S Collins EG Quinn LT Park CG Ferrans CE. Enriching the quality of cross-cultural instrument development through cognitive interviewing: Implications for nursing research. Jpn J Nurs Sci. (2020) 17:12301: 10.1111./jjns.12301 31721460

[18] Park H Sha MM Pan YL. Investigating validity and effectiveness of cognitive interviewing as a pretesting method for non-English questionnaires: findings from Korean cognitive interviews. Int J Soc Res Methodol. (2014) 17:643–58. 10.1080/13645579.2013.823002

[19] Yu ZY Jin YY Lor M. Evaluating culturally tailored strategies for implementing cognitive interviewing on edinburgh postnatal depression scale among Chinese immigrant women. J Transcul Nurs. (2020) 2:50437. 10.1177./1043659620950437 34455833

[20] Martin SL Birhanu Z Omotayo MO Kebede Y Pelto GH Stoltzfus RJ. I can't answer what you're asking me. Let me go, please: cognitive interviewing to assess social support measures in Ethiopia and Kenya. Field Meth. (2017) 29:317–32. 10.1177/1525822x17703393

[21] Mavhu W Langhaug L Manyonga B Power R Cowan F. What is 'sex' exactly? Using cognitive interviewing to improve the validity of sexual behaviour reporting among young people in rural Zimbabwe. Cult Health Sex. (2008) 10:563–72. 10.1080/13691050801948102 18649195

[22] Simwinga M Kumwenda MK Dacombe RJ Kayira L Muzumara A Johnson CC. Ability to understand and correctly follow HIV self-test kit instructions for use: applying the cognitive interview technique in Malawi and Zambia. J Int AIDS Soc. (2019) 22. ARTN e25253 10.1002./jia2.25253 30907496 PMC6432102

[23] Vreeman RC Nyandiko WM Ayaya SO Walumbe EG Inui TS. Cognitive interviewing for cross-cultural adaptation of pediatric antiretroviral therapy adherence measurement items. Int J Behav Med. (2014) 21:186–96. 10.1007/s12529-012-9283-9 23188670

[24] Johnson TP Pennell BE Stoop IAL Dorer B eds. Advances in Comparative Survey Methods. Multinational, Multiregional, and Multicultural Contexts (3MC). Hoboken, NJ 07030, USA: John Wiley and Sons, Inc (2019).

[25] Survey Research Centre,. Guidelines for Best Practice in Cross-Cultural Surveys. Full Guidelines. Institute for Social Research, University of Michigan (2016). Available online at: http://www.ccsg.isr.umich.edu/ (accessed April 23, 2019).

[26] Miller K Fitzgerald R Padilla JL Willson S Widdop S Caspar R. Design and analysis of cognitive interviews for comparative multinational testing. Field Meth. (2011) 23:379–96. 10.1177/1525822x11414802 29081719 PMC5646372

[27] Scott K Ummer O LeFevre AE. The devil is in the detail: reflections on the value and application of cognitive interviewing to strengthen quantitative surveys in global health. Health Policy Plann. (2021) 36:982–95. 10.1093/heapol/czab048 33978729 PMC8227989

[28] Colbert CY French JC Arroliga AC Bierer SB. Best practice versus actual practice: an audit of survey pretesting practices reported in a sample of medical education. J Med Edu Online. (2019) 24:3596 10.1080/10872019.(2981)1673596 31671286 PMC6830238

[29] Woolhouse M Ward M van Bunnik B Farrar J. Antimicrobial resistance in humans, livestock, and the wider environment. Philosoph Transact Royal Soc B-Biol Sci. (2015) 370:1670. ARTN 20140083/ 10.1098./rstb.2014.0083 25918441 PMC4424433

[30] Wieland B Dione M Alemu B Fevre E Grace D Omoya L. AMUSE Livestock, Version 2-Antimicrobial Use in Livestock Production: A Tool to Harmonize Data Collection on Knowledge, Attitude, Practices. ILRI (Nairobi, Kenya). (2019). Available online at: https://hdl.handle.net/10568/107443 (accessed June 23, 2022).

[31] Alemu B Amenu K Magnusson U Dohoo I Hallenberg GS Alemayehu G. Antimicrobial use in extensive smallholder livestock farming systems in Ethiopia: Knowledge, attitudes and practices of livestock keepers. Front Veter Sci. (2020) 7:55. 10.3389./fvets.2020.00055 32175334 PMC7055293

[32] Christine AW Dione M Okello L Magnusson U Wieland B. Antimicrobial use in smallholder pig production systems in Uganda. CGIAR Antimicrobial Resistance Hub Launch, Nairobi, Kenya: ILRI. (2019). Available online at: https://hdl.handle.net/10568/100287 (accessed February 22, 2019).

[33] Ndoboli D Lukuyu B Wieland B Grace D Roesel K. The use of antimicrobials to boost livestock productivity at Ugandan piggery and poultry farms and implications for public health. Paper presented at the Seventh All Africa Conference on Animal Agruculture Accra, Ghana. (2019). Available online at: https://hdl.handle.net/10568/106385” https://hdl.handle.net/10568/106385 (accessed July 2, 2019).

[34] Lee HS Wieland B Hung NV Magnusson U. Use of Veterinary Drugs in Livestock Production in Vietnam: Study Report on Survey in CRP Livestock targeted sites. Nairobi: ILRI (2019). Available online at: https://hdl.handle.net/10568/107441 (accessed June 23, 2022).

[35] Mohorko A Hiebec V. Degree of cognitive interviewer involvement in questionnaire pretesting on trending survey modes. Comp Human Behav. (2016) 62:79–89 10.1016/jchb03

[36] Pretty NJ Guijt I Thompson J Scoones I. A trainer's guide for participatory learning and action. IIED (1995). Available online at: https://pubs.iied.org/6021iied (accessed June 23, 2022).

