# Supplementary material for: “How Are My Age and Cows Related?” Cognitive Interviewing as a Tool to Pretest Survey Questions in Two Limited Resource Settings
Source: Front Vet Sci. 2022 Jul 8;9:833748. doi: 10.3389/fvets.2022.833748 (PMC9305482; doi:10.3389/fvets.2022.833748)
Supplement: Supplementary file 1 [file Data_Sheet_1.PDF]

Antimicrobial use in livestock production systems (AMUSE Livestock): Tool to harmonise data collection on knowledge, attitude and practices

This tool has been prepared by the Livestock Health flagship of the CRP LIVESTOCK

Barbara Wieland, Michel Dione, Biruk Alemu Gemeda, Eric Fevre, Delia Grace, Louis Omoya, Gunilla Ström, Elisabeth Lindahl, Ulf Magnusson

Antimicrobial resistance has been recognised as a major threats to public health. As a consequence more research is being conducted in this field, including research on use of antimicrobials in the agricultural sector, especially in livestock production. Data and knowledge of use of antimicrobials in low and middle income countries (LMIC) is scarce and no centralised reporting and recording systems exist for monitoring purposes.

Given the recent increase in numbers of research and development projects focussing on antimicrobials in livestock production, there is a need for a common tool to be used in different projects. Use the same questions for basic data collectionh in different projects will simplify collation of data across projects and production systems and potentially provide important information on trends of antimicrobial use and areas of particular concern.

This questionnaire was developed to investiagate key linkages in the AMR conceptual framework outlined below (adapted from Woolhouse et al, 2015):

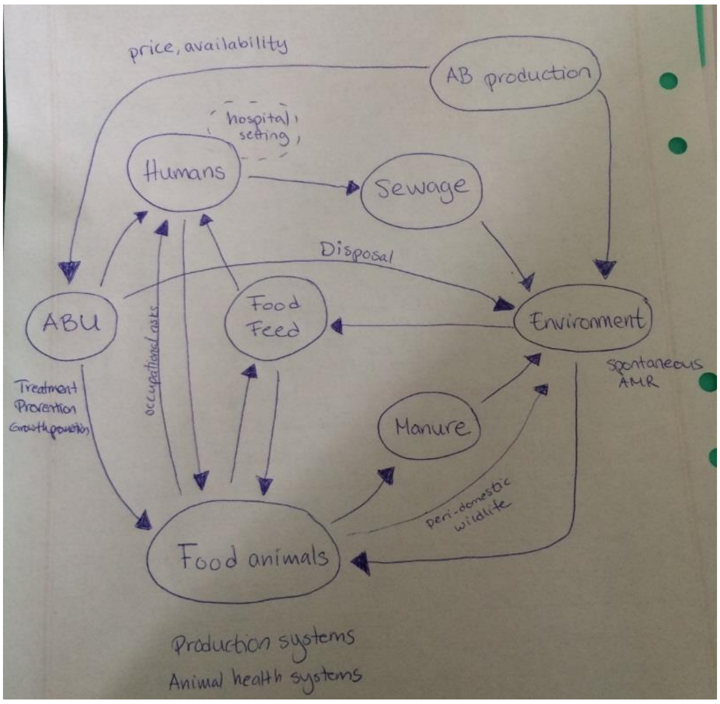

and is meant to provide a core set of questions that may ease compaisons of data from different regions of the world collected in different studies in different production systems.

Different projects have different purposes and objectives and thus may need additional questions. Therefore the questionnaire may then be expanded with other questions for more in depth study of particular aspects of AMU/AMR in the livestock sector or to fullfil other study objectives.

The AMUSE Livestock questionnaire has been tested in Ethiopia, Kenya and Uganda and from experience should not take more than 30 minutes to complete.

Check-list:

- ✓ Find out and document the broader context of access (including leagalisation related to) to animal health service and pharmaceuticals (incl antimicrobials) for the study site.
- ✓ Consider other surveys or national inventories that have been made at the study site.
- ✓ Always pre-teste the questionnaire on farmers.
- ✓ Consider data-collection format/layout
- ✓ Get pictures or samples of commonly used drugs and organise them into drug classes (for numbering see question 40).
- ✓ Consider complementing questionnaire by observational data (ideally photo), if so make this clear.
- ✓ Ensure ethical clearance (Helsinki declaration) for your study has been obtained including details on informed consent, purpose of study, guaranteed anonymity, feedback of information to community etc

The AMUSE Livestock KAP tool is available electronically (in ODK), for further information and to receive the necessary files, please contact Barbara Wieland at ILRI (b.wieland@cgiar.org).

**Criteria for selecting respondent: person who plays a major role in the management of livestock**<br>**INFORMATION ON ENUMERATION**	
1. Questionnaire ID	
2. Date of Survey (DD/MM/YYYY)	
3. Enumerator’s name (First Name and Last Name)	List of names of enumerators
4. Interview done via interpreter	☐1=yes<br>☐2=now
5. Enumerator’s sex	☐1=Male, (If list available then this should be automatically filed)<br>☐2=Female
6. Time interview started (HH:MM)	Will be automatically generated by the tablets
7. Time interview ended (HH:MM)	Will be automatically generated by the tablets
8. Consent received (signature on form if literate)	☐1=yes<br>☐2=no
**FARM BASICS AND LOCATION**	
9. District	List of all districts pre-coded
10. Sub-county	List of all sub counties pre-coded
11. Parish	List of all parishes pre-coded
12. Village	List of all village pre-coded
13. GPS Coordinates	Will be automatically generated by the tablets
**HOUSEHOLD DEMOGRAPHICS**	

14. Sex of the Household head	☐1=Male<br>☐2=Female
15. Sex of the respondent (if other than household head)	☐1=Male<br>☐2=Female
16. Age of respondent (years)	…………………
17. Role of the respondent in relation to livestock (multiple answers possible)	☐1 Management<br>☐2 Marketing<br>☐3 Owner<br>☐4 None<br>☐ 5 other
18. What is the main source of income for the household? (Mark one)	☐ 1 crop farming<br>☐ 2 cattle keeping<br>☐ 3 pig keeping (inc. sales)<br>☐ 4 small ruminant keeping<br>☐ 5 poultry keeping<br>☐ 6 salaried employment<br>☐ 7 self-employed-off farm<br>☐ 8 casual laboring<br>☐ 9 Boda-boda<br>☐ 10 other (specify)………………………………………….
19. Livestock contributes to	☐ 1 To half or more of the household's income<br>☐ 2 To less than half of the household's income<br>☐ 3 Does not contribute to the household income
20. What is the education level of the **respondent**?	☐ 1 Never went to school<br>☐ 2 Non-formal education (years)……………………..<br>☐ 3 Primary education (P1-P7)<br>☐ 4 Secondary school (S1-S6)<br>☐ 5 Vocational training (specify)………………………..<br>☐ 6 University degree (undergraduate)<br>☐ 7 Adult literacy
21. Do you have hired workers on the farm	☐ Yes 1<br>☐ No, family members only 2
**FARM CHARACTERISTICS**	
22. What livestock do you have? (filter question)	☐1Cattle (beef, dairy)<br>☐2Small ruminants<br>☐3Poultry<br>☐4Pigs<br>☐5Equine<br>☐6Camel
23. Who has the **main** responsibilty (for each species)	
☐ 1 Household head (man)	
☐ 2 Household head (woman)	
☐ 3 Joint responsibility (Couple	
☐ 4 Daughter	
☐ 5 Son	
☐ 6 Employee	
☐ 7 Other (specify)	
24. Herd flock size (mumber of animals for each species)	

a) Poultry	Number…………………….	
Equines	Number…..	
b) Cattle	Adult males (>2 years)	
	Adult females (>2 years)	
	Calves/heifers	
c) Pigs	Sows	
	Boars	
	Growers/fatteners	
	Piglets (<3 moths)	
d) Small ruminants	Males (>1 year)	
	Females (>1 year)	
	Young	
e) Camels		

25. Characteristics of livestock production systems (single choice/most common practice throughout the year)		
**1. PIGS**		☐ 1= free-range<br>☐ 2= tethered<br>☐ 3= housed
**2. POUL TRY**		☐ 1= free-range<br>☐ 2= housed
**3. Cattle**	☐Beef	☐1 = Zero grazing<br>☐2 = Fenced individual farm grazing<br>☐3 = Communal grazing<br>☐4 = Pastoral
	☐ Dairy	☐1 = Zero grazing<br>☐2 = Fenced individual farm grazing<br>☐3 = Communal grazing<br>☐4 = Pastoral
4. Small ruminants		☐1 = Zero grazing<br>☐2 = Fenced individual farm grazing<br>☐3 = Communal grazing<br>☐4 = Pastoral
5. Equines		☐1 = Zero grazing<br>☐2 = Fenced individual farm grazing<br>☐3 = Communal grazing<br>☐4 = Pastoral
6. Camels		☐1 = Zero grazing<br>☐2 = Fenced individual farm grazing<br>☐3 = Communal grazing<br>☐4 = Pastoral

26. Do you sell milk	☐ Yes 1<br>☐ No 2
	Throughout the year ☐

<table>
<tr><td rowspan="3">27. Which period of the year do you regularly sell milk? (multiple choices are allowed for the months)</td><td colspan="12">Seasonal (use calendar below) ☐</td></tr>
<tr><td>J</td><td>F</td><td>M</td><td>A</td><td>M</td><td>J</td><td>J</td><td>A</td><td>S</td><td>O</td><td>N</td><td>D</td></tr>
<tr><td></td><td></td><td></td><td></td><td></td><td></td><td></td><td></td><td></td><td></td><td></td><td></td></tr>
<tr><td>28. Do you sell eggs (multiple choices are allowed for the months)</td><td colspan="12">☐ Yes 1
☐ No 2</td></tr>
<tr><td rowspan="4">29. Which period of the year do you regularly sell eggs? (multiple choices are allowed for the months)for the months</td><td colspan="12">Throughout the year ☐</td></tr>
<tr><td colspan="12">Seasonal (use calendar below) ☐</td></tr>
<tr><td>J</td><td>F</td><td>M</td><td>A</td><td>M</td><td>J</td><td>J</td><td>A</td><td>S</td><td>O</td><td>N</td><td>D</td></tr>
<tr><td></td><td></td><td></td><td></td><td></td><td></td><td></td><td></td><td></td><td></td><td></td><td></td></tr>
<tr><td>30. Do you sell live animals ?</td><td colspan="12">☐ Yes 1
☐ No 2</td></tr>
</table>

<table>
<tr><td rowspan="18">31. If yes, which species? Indicate during which months of the year you sell live animals (multiple choices are allowed for the months)</td><td colspan="12">☐1 Pigs
Throughout the year ☐
Seasonal (use calendar below) ☐</td></tr>
<tr><td>J</td><td>F</td><td>M</td><td>A</td><td>M</td><td>J</td><td>J</td><td>A</td><td>S</td><td>O</td><td>N</td><td>D</td></tr>
<tr><td></td><td></td><td></td><td></td><td></td><td></td><td></td><td></td><td></td><td></td><td></td><td></td></tr>
<tr><td colspan="12">☐2 Poultry
Throughout the year ☐
Seasonal (use calendar below) ☐</td></tr>
<tr><td>J</td><td>F</td><td>M</td><td>A</td><td>M</td><td>J</td><td>J</td><td>A</td><td>S</td><td>O</td><td>N</td><td>D</td></tr>
<tr><td></td><td></td><td></td><td></td><td></td><td></td><td></td><td></td><td></td><td></td><td></td><td></td></tr>
<tr><td colspan="12">☐3 Cattle
Throughout the year ☐
Seasonal (use calendar below) ☐</td></tr>
<tr><td>J</td><td>F</td><td>M</td><td>A</td><td>M</td><td>J</td><td>J</td><td>A</td><td>S</td><td>O</td><td>N</td><td>D</td></tr>
<tr><td></td><td></td><td></td><td></td><td></td><td></td><td></td><td></td><td></td><td></td><td></td><td></td></tr>
<tr><td colspan="12">☐4 Small ruminants
Throughout the year ☐
Seasonal (use calendar below) ☐</td></tr>
<tr><td>J</td><td>F</td><td>M</td><td>A</td><td>M</td><td>J</td><td>J</td><td>A</td><td>S</td><td>O</td><td>N</td><td>D</td></tr>
<tr><td></td><td></td><td></td><td></td><td></td><td></td><td></td><td></td><td></td><td></td><td></td><td></td></tr>
<tr><td colspan="12">☐5 Equines
Throughout the year ☐
Seasonal (use calendar below) ☐</td></tr>
<tr><td>J</td><td>F</td><td>M</td><td>A</td><td>M</td><td>J</td><td>J</td><td>A</td><td>S</td><td>O</td><td>N</td><td>D</td></tr>
<tr><td></td><td></td><td></td><td></td><td></td><td></td><td></td><td></td><td></td><td></td><td></td><td></td></tr>
<tr><td colspan="12">☐6 Camel
Throughout the year ☐
Seasonal (use calendar below) ☐</td></tr>
<tr><td>J</td><td>F</td><td>M</td><td>A</td><td>M</td><td>J</td><td>J</td><td>A</td><td>S</td><td>O</td><td>N</td><td>D</td></tr>
<tr><td></td><td></td><td></td><td></td><td></td><td></td><td></td><td></td><td></td><td></td><td></td><td></td></tr>
</table>

MANAGEMENT OF MANURE, FEED AND WATER						
32. Manure management (by species), tick the **most common** option for each species (single choice).						
Activity	1.Cattle	2.Small ruminants	3. Equines	4. Poultry	5. Pigs	6. Camel
a. Leave on farm, do nothing						
b. Discard into environment						
c. Open air						
d. Used as fertilizer						
e. Use for fuel (incl. biogas)						
f. Sold for cash						
g. Taken by other farmers						
h. Other (specify)						
33. Feed products used per species (mulpile answers per species possible) , tick						
Type of feed	1.Cattle	2.Small ruminants	3. Equines	4. Poultry	5. Pigs	6. Camel
Pasture/scavenging						
Waste (household/restaurant, etc)						
grains/crop residues						
Feed mixed at farm						
Commercial/pre-mix						
Other						

ANIMAL HEALTH AND DISEASE PREVENTION							
34. What was the **main animal disease problem** during the last 12 months (one disease per species)-if the farmers says FEVER, probe for more clinical signs because fever is common for most diseases							
Clinical signs	1.Cattle	2.Goats	3. Sheep	4. Poultry	5. Pigs	6. Equines	7. Camel
a) Respiratory							
b) Digestive tract/intestinal							
c) Reproductive							
d) Mastitis							
e) Sudden death							
f) Skin disease/wounds							
g) External parasites							
h) Neurologic signs							
i) Other							
j) no disease problem							

35. Have any animals been sick in the last 2 weeks?	☐ 1 YES<br>☐ 2 NO
36. If yes, which animal and kind of disease?	Optional: Use a table of clinical signs by species and key diseases in annex

Clinical signs	1.Cattle	2.Goats	3. Sheep	4. Poultry	5. Pigs	6. Camel
a) Respiratory						
b) Digestive tract/ intestinal						
c) Reproductive						
d) Mastitis						
e) Sudden death						
f) Skin disease						

g) neurologic signs						
h) Other						

37. Was the disease diagnosed other than by yourself?	☐ 1 YES ☐ 2 NO
38. If yes, by whom?	☐ 1 Traditional healer<br>☐ 2 community animal health worker<br>☐ 3 private veterinarian (Diploma, BVM)<br>☐ 4 official (governmental) veterinarian<br>☐ 5 Other (specify)……………………………

39. What do you do in response to diseases problems? (refer to the recent disease problems mentionned above)

Activity	1.Cattle	2.Goats	3.Sheep	4.Poultry	5.Pigs	6.Camel
a) Use traditional medicine						
b) Use medicine from the veterinary drug store (self-bought)						
c) Consult traditional healer						
d) Consult community animal health worker						
e) Consult private veterinarian						
f) Consult Government veterinarian						
g) Vet applied/left drugs						
h) Other, please specify						

40. From the drug categories shown (provide photographs of common veterinary drugs for each of the drug class), how often (how many times) have you used them for the different species in the last 2 months. Alternatively ask to see packaging of commonly used drugs and classify according to list below.

Drug	1 Cattle	2 Small ruminants	3 Equines	4 Poultry	5 Pigs	6 Camels
1 Vaccines						
2 Antihelmintics (Albendazol, etc.)						
3 Arachnidicides (ectoparasites)						
4 Tetracyclines						
5 Sulphonamides						
6 Penicillin (and combinations with Penicillin)						
7 Fluoroquinolones						
8 Macrolides						
9 Aminoglycosides						
10 Other antibiotics (specify)						
11 Vitamins/Iron supplements						
12 Other drugs (specify)						

41. Do you have any particular means to protect animals from disease?	☐ 1 YES ☐ 2 NO
42. If yes, how?	

a) Activity	1.Cattle	2.Goats	3.Sheep	3.Poultr y	4.Pigs	5.Fish	6.Camel s
b) Fencing							
c) Not mixing with other herd/flock							
d) Special feed							
e) Vet drugs (incl vaccine)							
f) Do nothing							
g) Other, specify							

**ANIMAL HEALTH SERVICES**	
43. Does the farm have access to professional animal health services?	☐ Yes 1<br>☐ No 2
44. If your farm access to animal health services, which ones?	☐1 State or government:<br>☐a) Fully trained veterinarian (BSc level)<br>☐b) Paraveterinarian<br>☐c) Other animal health care provider;<br>☐d) Don't know the training or qualification<br>☐2 Private full time animal health worker:<br>☐a) Fully trained veterinarian (BSc level)<br>☐b) Paraveterinarian<br>☐c) Other animal health care provider;<br>☐d) Don't know the training or qualification<br>☐3 Both state/government and private<br>☐a) Fully trained veterinarian (BSc level)<br>☐b) Paraveterinarian<br>☐c) Other animal health care provider;<br>☐d) Don't know the training or qualification<br>☐4 Other (specify)……………………………………………….
45. If you have access to animal health services, do the animal service include laboratory testing?	☐ Yes 1<br>☐ No 2
46. If you have access to laboratory services, do you use it?	☐ 1 Yes, when needed<br>☐ 2 Rarely<br>☐ 3 No

47. If yes, for diagnosis in which species?

1.Cattle	2.small ruminants	3.Equines	4.Poultry	5.Pigs	6. Camels	

48. If you don't use them, why?	☐ 1 Not available<br>☐ 2 Not efficient<br>☐ 3 Too expensive<br>☐ 4 Would like more<br>☐ 5 Other (specify)……………………….
49. Is the farm involved in a regular animal health service program, like vaccination campaign etc run by government and/or NGO?	☐ 1 Yes<br>☐ 2 No

50. If yes to above, please name	……………………………
51. Do you access pharmaceuticals/veterinary drugs?	☐ 1 Yes<br>☐ 2 No
52. If yes, which kind of pharmaceuticals/veterinary drugs have you used in the last 4 weeks? (LIST PER SPECIES, PHOTOGRAPH)	List them:……………………………..

VETERINARY DRUG USE: THE FOLLOWING QUESTIONS FOR EACH SPECIES PRESENT IN THE FARM (ONE DRUG)	
53. Which of the drugs is the most commonly used? (pictures or drug samples) (refer to Q51)	DROP DOWN LIST WITH VET DRUGS
54. Why do you use this drug?	☐ 1 Disease prevention<br>☐ 2 Treatment sick animal<br>☐ 3 Fattening<br>☐ 4 Other (specify)
55. Via which channel do you access this pharmaceuticals/veterinary drugs	☐ 1 Private vet<br>☐ 2 Public/official vet<br>☐ 3 Animal health worker<br>☐4 Veterinary drug store<br>☐ 5 From human pharmacies<br>☐ 6 At markets<br>☐ 7 Feed providers<br>☐ 8 Other farmers<br>☐ 9 Via NGOs☐ 10 Other (specify)
56. To which animals do you give the drug?	☐ All of the same species<br>☐ Sick animals only<br>☐ Sick and in contact animals<br>☐ Before selling an animal<br>☐ Animals newly introduced into herd<br>☐ All animals in household
57. How long do you use the drug?	☐ As advised<br>☐ Until animal(s) cured<br>☐ Until package empty<br>☐ As long as I can afford<br>☐ One time treatment<br>☐ Continiously over extended period<br><br>Estimated average number days……………….
58. Who adminster the drug?	☐ 1 Myself<br>☐ 2 Vet<br>☐ Other (specify)………..
59. How is the drug applied/given?	☐ 1 Injection<br>☐ 2 Oral<br>☐ 3 with feed<br>☐ 4 with water<br>☐ 5 on skin

	☐ 6 other (specify)………

60. Do you get advice how to use the vet drugs?	☐ Yes 1<br>☐ No 2
61. If yes to above, via which channel?	☐ 1 from the veterinarians<br>☐ 2 from the animal health worker<br>☐ 3 from pharmacies or markets<br>☐ 4 from other farmers<br>☐ 5 via the feed provider<br>☐ 6 from the package/label of he pharmaceutical<br>☐ 7 Other, state who………<br>☐ 8 No, own judgment
62. When using veterinary drugs, whose instructions (kind, dose, length of treatment) do you follow:	☐1 The Veterinarian's<br>☐2 The animal health worker's<br>☐3 The pharmacy's<br>☐4 The feed company's<br>☐5 Other farmer's<br>☐6 My own judgement<br>☐7 Other`s(specify)……………………………….

**USE OF ANTIBIOTICS**	
63. What does vaccination do? (multiple answers possible)	☐1 Cure sick animals<br>☐2 Prevent animals from becoming sick<br>☐3 Cure sick animals and prevent animals from becoming sick<br>☐4 Fattening
64. What do antibioticls do? (multiple answers possible)	☐1 Cure sick animals<br>☐2 Prevent animals from becoming sick<br>☐3 Cure sick animals and prevent animals from becoming sick<br>☐4 Fattening
65. If the subject understands what antibiotics (option 1 and 3) are based on the two questions above continue as follows:	
66. Do you consume milk, from animals who were just treated with antimicrobials.	☐ 1 Yes<br>☐ 2 No
67. If No to the above, for how long time should those products be avoided (open) (in days)	………………………………
68. Do you consume eggs from animals who were just treated with antimicrobials.	☐ 1 Yes<br>☐ 2 No

69. If No to the above, for how long time should those products be avoided (open) (in days)	………………………………
70. Do you consume meat from animals who were just treated with antimicrobials.	☐ 1 Yes<br>☐ 2 No
71. If No to the above, for how long time should those products be avoided (open) (in days)	………………………………

72. Have you experienced situations where drugs did not work	Yes, frequently<br>Yes, sometimes<br>No, never

73. If you have experience with drug failure (YES to Q71), which drugs did not work?

Drug on pictures shown	1.Cattle	2.Poultry	3.Pigs	4.Small ruminants	5.Equine	6.Camels
Drug list						
Drug list						
Drug list						

74. If the veterinary drug do not work, do you know why?	…………………………………………………………
75. What do you do with expired veterinary drugs?	☐1 Dispose off<br>☐2 Return to pharmacy<br>☐3 Give to other farmer<br>☐4 Use for intended treatment<br>☐5 Nothing<br>☐6 Other (specify)
76. **FOR EACH SPECIES:** What was your total expenditure in drugs during the last year in local currency (put 0 if none)	☐1 Dewormer […………………]<br>☐2 Vaccination […………………]<br>☐3 Antibiotics […………………]<br>☐4 Acaracides […………………]<br>☐5 Vitamins

NB: Note that the GIS may take some time to load approximately 5 minutes however they work

List of annexes

1. List of pictures of drugs, organised into drug classes as specified in question 40.
2. List of clinical signs by syndrome
3. Groups drug used by de-wormers, vaccines, AB, vitamins
4. List of social events and festive seasons with the months they happen
